# Case Report: From trivial trauma to fulminant septic shock: multidisciplinary rescue of *Vibrio vulnificus* necrotizing fasciitis via a seven-stage surgical protocol with limb salvage

**DOI:** 10.3389/fmed.2026.1714153

**Published:** 2026-01-22

**Authors:** Tianen Pan, Xin Zhuang, Lina Xiang

**Affiliations:** 1 Department of Infectious Diseases, Pingyang Hospital of Traditional Chinese Medicine Zhejiang Chinese Medical University, Wenzhou, China; 2 Department of Hematology, The First Affiliated Hospital of Wenzhou Medical University, Wenzhou, China; 3 Department of Emergency, The First Affiliated Hospital of Wenzhou Medical University, Wenzhou, China

**Keywords:** multidisciplinary rescue, multiple organ dysfunction syndrome (MODS), necrotizing fasciitis, septic shock, *Vibrio vulnificus*

## Abstract

**Objective:**

To report a successful case of an inland seafood vendor who developed *Vibrio vulnificus* necrotizing fasciitis complicated by septic shock following a minor calf abrasion, and to explore its special epidemiological implications and key points for standardized management.

**Case summary:**

A 46-year-old male seafood vendor (hospitalized from July 3 to 10 August 2025) presented on post-injury day 7 with fulminant necrotizing fasciitis, septic shock, and multiple organ dysfunction syndrome. *Vibrio vulnificus* was identified by wound culture and metagenomic sequencing. Management included early combination antibiotics, ICU organ support, and seven sequential surgical interventions. The patient was successfully weaned from mechanical ventilation and extubated after 25 days of ICU care, and discharged on hospital day 30 with satisfactory wound healing.

**Conclusion:**

This case alerts that high inoculum exposure due to cold-chain disruption can prolong the incubation period of *V. vulnificus* infection to 7 days, transcending traditional epidemiological boundaries. Successful management depended on early fasciotomy and strict adherence to standardized treatment protocols. Mandatory wound monitoring for high-risk occupational populations should become a new priority in public health prevention and control.

## 1 Introduction

*Vibrio vulnificus* is a halophilic (0.5%–3.0% NaCl), mesophilic (optimal temperature 26–37 °C), facultatively anaerobic Gram-negative bacillus of the family Vibrionaceae, appearing as comma-shaped, straight rods, or coccobacilli under microscopy (1). This bacterium is ubiquitous in warm seawater and seafood products. *Vibrio vulnificus* septicemia (Vv-S) constitutes an acute critical illness; once it progresses to necrotizing soft tissue infection (Vv-NSTI), mortality exceeds 50% (2, 3). Cases predominantly occur from March through November, peaking in summer, and are primarily observed in coastal regions; disease onset typically manifests 12–72 h post-infection (4). The principal clinical syndromes include: (1) foodborne primary septicemia, commonly triggered by consumption of raw oysters or other shellfish; (2) wound-associated necrotizing fasciitis, wherein bacteria invade through skin breaches exposed to seawater or contaminated seafood, with mortality rates of 50%–60% (5). Susceptible hosts encompass individuals with chronic liver disease, alcoholism, hemochromatosis, diabetes mellitus, or other immunocompromised conditions; among these, case-fatality rates in hepatic disease patients surpass 50% (6). Reported cases of *V. vulnificus* infection in China have been increasing annually. To enhance clinical awareness and diagnostic-therapeutic standards, this article presents a successfully managed case of *V. vulnificus* bloodstream infection complicated by septic shock following leg trauma, admitted in July 2025.

## 2 Case introduction

The patient was a 46-year-old male seafood vendor with a history of chronic hepatitis B virus infection; hyperglycaemia was noted on admission. On 3 July 2025 he collided with an electric scooter loaded with seafood while restocking, sustaining a mung-bean-sized wound on the left calf. After self-administering povidone-iodine he sought no further care. The lesion subsequently became painful and ulcerated, accompanied by swelling, increased local temperature, and scant serous discharge of the lower limb. On day 7 post-injury (July 10), the patient developed high-grade fever with a peak self-measured temperature of 39 °C at home, and subsequently presented to our emergency department.

**FIGURE 1:**
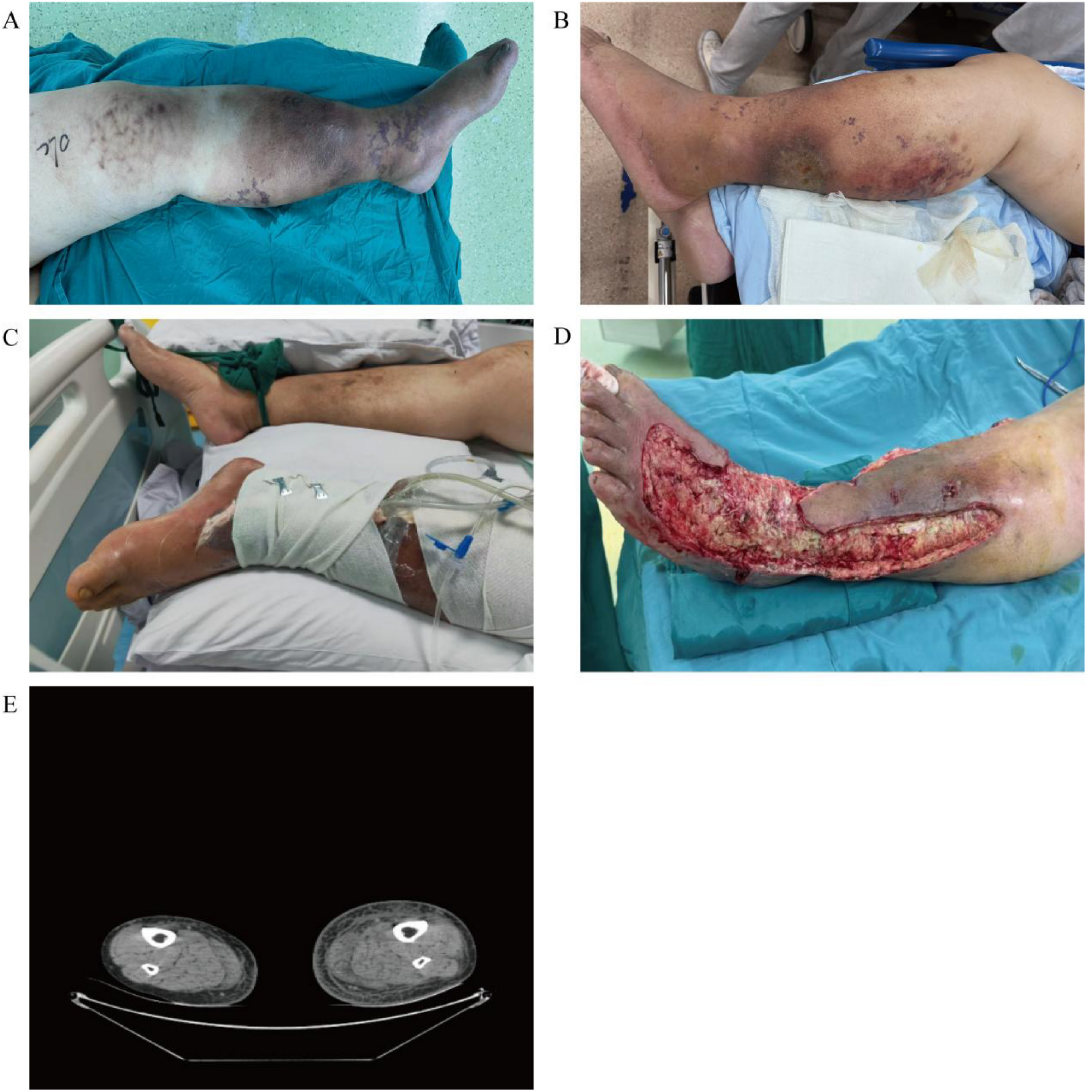
**(A,B)** On the day of admission, the patient’s left lower limb exhibited erythema and swelling; a small, exudative wound was visible on the posterior calf, and extreme compartment tension had led to the formation of tense bullae. **(C)** Post-operative photograph after the second procedure: VSD, escharotomy and decompressive fasciotomy of the lower-limb compartments. **(D)** Intra-operative view during the third procedure: pedicled flap transfer, escharotomy and VSD. Extensive skin necrosis extended from the mid-calf to the dorsum of the foot; bilateral relaxing incisions exposed posterior, dorsal, medial and lateral areas of eschar-covered necrosis, with underlying fascia and adipose tissue largely devitalised. **(E)** On the day of admission, CT revealed multifocal soft-tissue swelling and exudation involving the left knee, lower leg and ankle.

Examination revealed an erythematous, indurated left lower extremity with a septic posterior-calf wound, extreme compartment tightness, and multiple tension bullae (Figures 1A, B). Vital signs: hazy consciousness, blood pressure 138/75 mmHg, heart rate 126 beats/min, respiration 37 beats/min, body temperature 39.8 °C, oxygen saturation 96% under nasal cannula 3 L/min oxygen intake. Laboratory values included white-cell count 10.12 × 10^9^/L, C-reactive protein 30.7 mg/L, procalcitonin 33 ng/mL, and lactate 2.5 mmol/L. Computed tomography (CT) showed diffuse soft-tissue swelling and fluid tracking from knee to ankle (Figure 1E). The clinical diagnosis includes septic shock accompanied by infectious multiple organ dysfunction syndrome (MODS), necrotizing fasciitis of the left lower leg, and osteofascial compartment syndrome of the left lower limb. The patient underwent oral tracheal intubation with assisted ventilation (SpO_2_: 85.2%, pH: 7.195) and received linezolid injections at 0.6 g every 12 h, in conjunction with imipenem and cilastatin injections at 1 *g* every 6 h for infection control. Additionally, m-hydroxylamine was administered to elevate blood pressure, along with fluid replacement and other symptomatic treatments.

Following multidisciplinary consultation, to halt infection progression, the first emergency surgery was performed on hospital day 2 (day 8 post-injury, July 11) under general anesthesia, comprising allogeneic skin grafting, allograft preparation, wound vacuum sealing drainage (VSD), and lower limb fasciotomy for decompression.

Pathological examination revealed that the “subcutaneous tissue of the left lower leg” exhibited deteriorated fibrous adipose tissue, with infiltration of inflammatory cells predominantly consisting of neutrophils within the fat lobule septa (Table 1). Post-operatively, the patient was transferred to the intensive care unit. Critical illness was reflected in the following laboratory values: creatinine 238 μmol/L, Alanine aminotransferase (ALT) 163 U/L, aspartate aminotransferase (AST) 216 U/L, white blood cell (WBC) 10.21 × 10^9^/L, C-reactive protein (CRP) 346.5 mg/L, procalcitonin > 100 ng/mL, and lactate 5.2 mmol/L. Echocardiography revealed globally reduced left ventricular wall contractility with moderate systolic dysfunction; ejection fraction (EF) was 35%. The composite diagnosis was necrotising fasciitis of the left calf, left lower-limb compartment syndrome, sepsis, septic shock, infective multiple-organ-dysfunction syndrome, stage 3 acute kidney injury, cardiac insufficiency, severe acute respiratory distress syndrome, and hepatic dysfunction. Bundled therapy was promptly initiated, including bedside continuous renal replacement therapy (CRRT), wound irrigation with gentamicin combined with chlorhexidine acetate solution, VSD, aggressive fluid resuscitation and vasopressor support for shock (norepinephrine combined with dobutamine), and other symptomatic measures. On hospital day 3 (9 days post-injury, July 12), cultures of both wound exudate and tissue specimens were positive for *Vibrio vulnificus*. Next-generation sequencing (NGS) performed on hospital day 4 (10th day post-injury, July 13) identified *Vibrio vulnificus* and hepatitis B virus, with a sequence count of 14 and a relative abundance of 28%. Susceptibility testing by the Kirby–Bauer (KB) disk-diffusion and minimum inhibitory concentration (MIC) methods demonstrated that the isolate was susceptible to aminoglycosides, fluoroquinolones, third- and fourth-generation cephalosporins, and carbapenems.

**TABLE 1:** Chronological summary of seven operative interventions: dates, procedures, and intra-operative findings.

Date (2025)	Procedure	Key intra-operative observations
11 July Hospital day 2	Allogeneic skin grafting, allograft preparation, VSD, fasciotomy	Left lower limb diffusely erythematous and edematous; extensive infected area on posterior calf with ecchymosis and minimal exudate; extreme compartment pressure; medial fasciotomy revealed abundant devitalized tissue.
14 July Hospital day 5	VSD, escharectomy, fasciotomy	After medial release, lateral compartments remained tense; scattered haemorrhagic bullae; circumferential skin necrosis covered by eschar; underlying fascia and fat markedly distorted (Figure 1C).
17 July Hospital day 8	Pedicled flap transfer, escharectomy, VSD	Bilateral calf–foot fasciotomies exposed persistent necrotic skin and eschar dorsally and posteriorly; gross architectural distortion of fascia and fat; posterior thigh erythematous with mildly elevated tension (Figure 1D).
21 July Hospital day 12	Escharectomy, pedicled flap transplantation, VSD	Residual necrotic tissue still evident circumferentially below the knee; medial thigh incision appeared viable. Pathology: suppurative necrotizing fasciitis.
24 July Hospital day 15	Pedicled flap transfer, VSD	Moderate necrotic debris remained on posterior calf, dorsum and aspects of the leg; previously sutured medial thigh wound healing well.
28 July Hospital day 19	Escharectomy, excisional skin debridement, VSD	Necrotic tissue burden unchanged; medial thigh donor site continued to epithelialize satisfactorily.
06 August Hospital day 28	Pedicled flap transfer, allogeneic skin grafting, allograft preparation	Granulation tissue now dominated the wound bed, which was bright red with scant yellow exudate; minimal bone exposed at medial malleolus; yellow necrotic patches visible beneath posterior skin; thigh incision fully healed.

On hospital day 2 (post-injury day 8, July 11), the patient was transferred to the ICU and empirical antimicrobial therapy was promptly initiated with linezolid 600 mg every 12 h plus imipenem-cilastatin 1 g every 6 h to cover suspected Gram-positive wound pathogens. After culture results became available, the regimen was adjusted to levofloxacin 500 mg once daily combined with imipenem-cilastatin 1 g every 6 h targeting *Vibrio vulnificus*. On hospital day 13 (post-injury day 19, July 22), the patient developed frequent premature ventricular contractions with significant QT interval prolongation, attributed to levofloxacin. Given decreasing inflammatory markers and stabilized body temperature, intravenous levofloxacin was discontinued.

**FIGURE 2:**
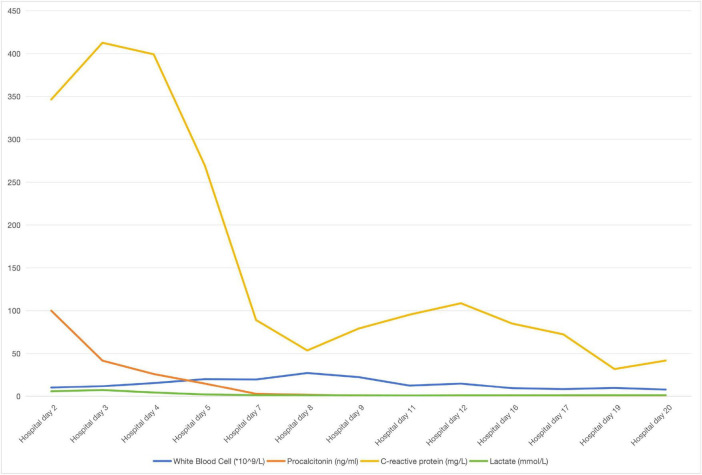
Temporal trend of white blood cell count, procalcitonin, C-reactive protein and lactate.

During hospital days 5–19 (14–28 July 2025), a 46-year-old male underwent five serial debridement procedures for progressive necrotizing soft-tissue infection of the left lower extremity (Table 1). The patient subsequently regained full consciousness with marked improvement in respiratory function. Systemic inflammation resolved, as evidenced by sequential declines in leukocyte count, procalcitonin, C-reactive protein, and lactate (Figure 2). Hepatic transaminases and renal indices normalized, while N-terminal pro-B-type natriuretic peptide (NT-proBNP) decreased, reflecting cardiac recovery. Myocardial injury biomarkers, including CK-MB, high-sensitivity troponin T, myoglobin, and creatine kinase, decreased in parallel. Point-of-care echocardiography demonstrated an ejection fraction of 63.7% with stable hemodynamics. During continuous renal replacement therapy (CRRT), the platelet count increased; however, repeated intraoperative bleeding caused modest reductions in hemoglobin concentration and erythrocyte count. After achieving clinical stability, the endotracheal tube and dialysis catheter were removed, and the patient was transferred from the ICU to the general ward on hospital day 17. On hospital day 28 (34 days after injury, August 6), the patient underwent the seventh operation (Table 1). The patient was discharged on hospital day 32 (38 days post-injury, August 10th) with the allogeneic skin graft *in situ* and no detectable subcutaneous fluid collection.

**FIGURE 3:**
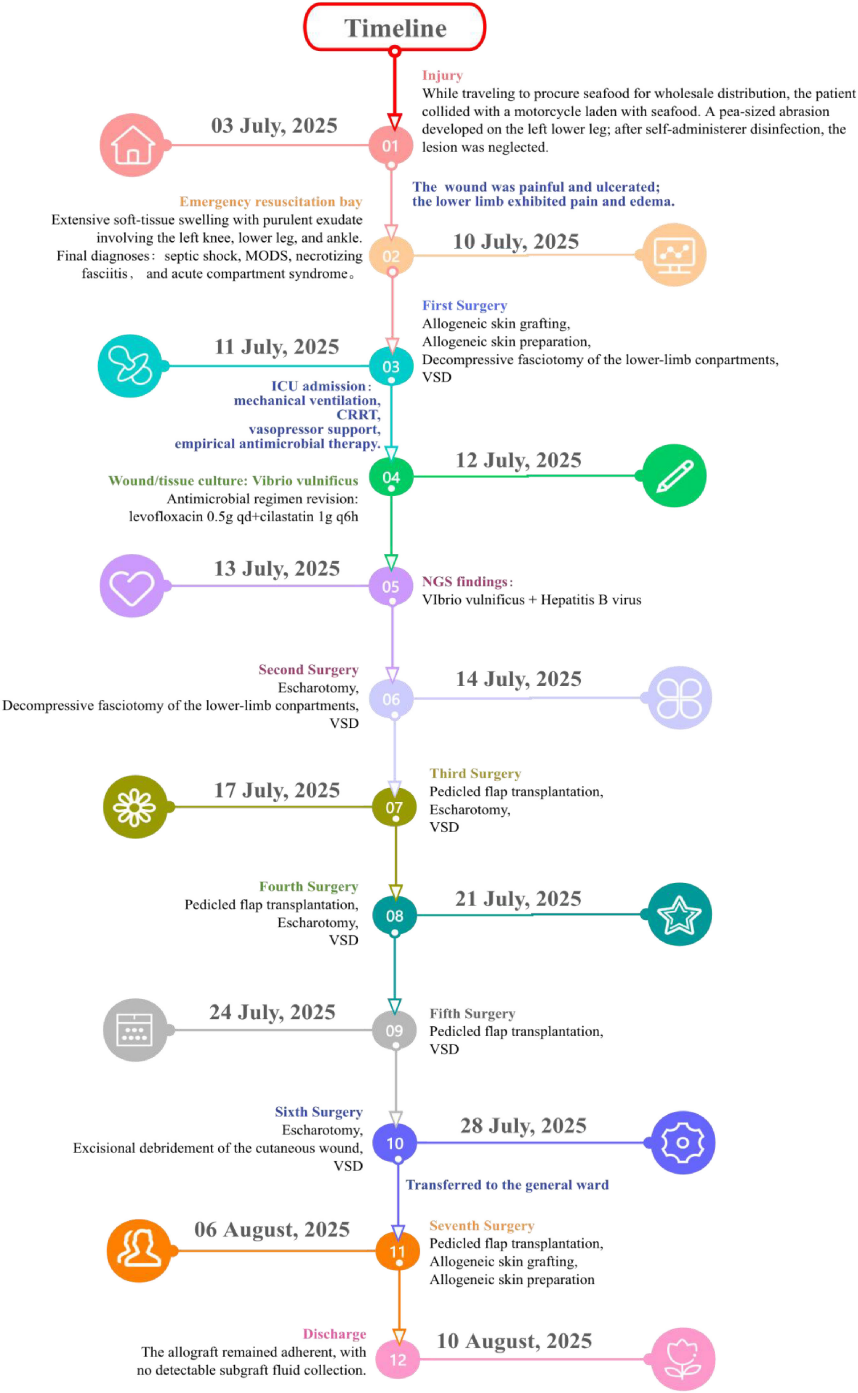
Treatment timeline from injury to discharge.

The complete care timeline, from admission to discharge, is summarized in Figure 3. At four months post-discharge, follow-up showed the grafted skin to be pink and viable without subcutaneous haematoma or pus; the donor site was partially re-epithelialised and dry. The patient has now progressed to ambulatory rehabilitation.

## 3 Discussion

*Vibrio vulnificus* proliferates exponentially in seawater and seafood at temperatures above 18 °C and enters dormancy at temperatures below 5 °C (7). Global warming has expanded its range from traditional tropical-subtropical coastal zones to temperate regions and even inland areas (8, 9). Unlike previous reports focusing on seawater exposure or oyster consumption (10), current evidence indicates that inland cold-chain transport and seafood processing facilities may serve as cryptic pathogen reservoirs, thereby expanding traditional epidemiological boundaries (11–13). Disease progression is exceptionally rapid, with extensive tissue damage, multiple-organ dysfunction, and underlying chronic hepatitis B, constituting a “high-exposure plus high-risk host” scenario (14–16). Through early fasciotomy, serial debridement, combination antibiotic therapy, continuous renal replacement therapy, and respiratory support, our patient achieved survival with limb preservation and functional recovery—an outcome superior to comparable severe cases (17–20).

This case exhibited a notably prolonged 7-day incubation period from minor abrasion to fulminant sepsis, significantly exceeding the typical latency reported for *Vibrio vulnificus* wound infections in the literature. Potential etiologies include: first, delayed bacterial inoculation—whereby the initial wound merely served as a portal of entry, with actual colonization occurring later through repeated handling of contaminated seafood during the patient’s daily work. This “intermittent exposure” pattern is distinctive to seafood handlers. Second, subclinical progression, where early localized infection may have been partially suppressed by self-administered povidone-iodine, delaying systemic dissemination until bacterial load reached a critical threshold. Third, host immunosuppression, as chronic hepatitis B combined with hyperglycemia may have blunted initial inflammatory responses, thereby masking early infection signs. Rather than diminishing credibility, this atypical feature underscores the critical importance of detailed occupational history in diagnosing occult *Vibrio vulnificus* infections.

The bacterium’s multiple toxins [MARTX (21), RtxA (22), hemolysin VvhA (23), etc.] synergize with the LPS-TLR4 axis to trigger a cytokine storm (24). The polysaccharide capsule masks complement C3 deposition and blocks opsonophagocytosis (25). Its iron acquisition mechanisms (such as siderophore and heme uptake systems regulated by the Fur–TonB system) are primarily induced under iron-limited conditions to efficiently chelate iron from the host (12, 26). Chronic hepatitis B accompanied by hyperglycemia provided a “double hit” of immunocompromise and iron metabolism dysregulation in this case (27). In this patient, chronic hepatitis B with hyperglycemia created a susceptible environment; portal hypertension not only drove bacterial translocation, but its associated alterations in iron metabolism also increased iron availability in host serum and tissues, thereby significantly enhancing bacterial iron acquisition and proliferation, accelerating toxin release and disease progression.

The patient’s long history of seafood-related work without prior infection demonstrates that disease onset is determined by a pathogen load threshold rather than mere exposure frequency. During the post-harvest transport phase, if seafood is removed from the cold chain (> 15 °C), *Vibrio vulnificus* can proliferate to > 10^5^ CFU/g within 24 h (13), far exceeding the defense threshold of healthy skin. Inland vendors are exposed to such high-concentration “terminal exposure sources” rather than low-concentration environmental seawater. This expands traditional epidemiological boundaries—risk is no longer limited to geographical coastlines but depends on cold chain integrity and biosafety at terminal processing stages. We recommend establishing mandatory temperature monitoring and worker wound reporting systems in inland seafood wholesale markets to interrupt this novel transmission chain.

The classical triad of wound-associated *Vibrio vulnificus* infection comprises pain disproportionate to cutaneous findings, haemorrhagic bullae, and rapidly advancing cellulitis or necrotising fasciitis (19). In the present case this “pain–bullae–progression” triad appeared on day 5 post-injury; together with a LRINEC score of 9 and CT-confirmed subcutaneous gas, necrotising fasciitis was highly probable. Notably, initial blood pressure remained normal, underscoring the occult nature of early shock. The 12-h interval in which serum lactate rose sharply represented the critical window for initiating the “golden 6-h” resuscitation bundle.

Metagenomic sequencing of wound exudate identified 14 *Vibrio* reads (28% relative abundance), providing definitive microbiological confirmation (28, 29). The isolate was fully susceptible to carbapenems, third-/fourth-generation cephalosporins, quinolones, and aminoglycosides, excluding antimicrobial resistance. Pathogen confirmation was achieved within 48 h of admission, permitting prompt targeted therapy. CDC guidelines recommend a third-generation cephalosporin plus a tetracycline as first-line therapy (30, 31); the 2024 Chinese *V. vulnificus* guidelines adjusted this to imipenem–cilastatin plus levofloxacin.

Multicentre studies have identified fasciotomy within 6 h as an independent predictor of survival (32, 33). The patient followed a three-stage protocol—“emergent decompression, serial debridement, functional reconstruction”—with operations every 2–3 days, combined with VSD and histological assessment to ensure clear infection margins and complete removal of necrotic tissue. The allogeneic skin graft achieved primary take without seroma or secondary infection, establishing the basis for limb salvage and functional recovery.

This inland seafood vendor—with no direct seawater contact—highlights refrigerated transport and seafood-handling environments as cryptic sources. Climate change and expanding cold-chain logistics are shifting the endemic boundary northward. Clinicians should therefore broaden epidemiological enquiries and maintain heightened vigilance among seafood workers, cold-chain employees, and market vendors. The episode reaffirms that seafood handlers with chronic liver disease, Hyperglycemia or immunosuppression embody a “high-exposure plus high-risk host” duality. Even trivial abrasions must be regarded as potential portals of entry: immediate irrigation, povidone-iodine disinfection, and 48-h clinical observation are mandatory. When primary-care facilities encounter the summer–autumn triad of “excruciating pain–haemorrhagic bullae–rapid progression,” TCBS culture should be ordered immediately and a multidisciplinary closed-loop protocol—“emergency fasciotomy + combination antibiotics + organ support”—initiated without delay. Nevertheless, this study has several inherent limitations: as a single-case report, it cannot establish causality or therapeutic efficacy, and our observations require validation through multicenter cohort studies.

## 4 Conclusion

*Vibrio vulnificus* necrotizing fasciitis can progress to septic shock and MODS within hours. This case demonstrates that adherence to the principles of early recognition, emergent debridement, combination antimicrobial therapy, organ support, and staged functional reconstruction may be life- and limb-saving. Clinicians should remain vigilant for the possibility of “minor trauma, major catastrophe,” particularly among seafood handlers, patients with chronic liver disease, and immunocompromised hosts.

## Data Availability

The original contributions presented in this study are included in this article/supplementary material, further inquiries can be directed to the corresponding author.
